# Sociodemographic and Clinical Profiles of Patients Admitted in Tertiary Level Pediatric Hospital of Nepal: An Observational Study

**DOI:** 10.31729/jnma.8911

**Published:** 2025-03-31

**Authors:** Anil Kumar Shrestha, Sushil Gyawali, Bal Mukunda Basnet, Santosh Adhikari, Sobi Lal Maharjan, Pujash Karmacharya, Deepak Raj Paudel

**Affiliations:** 1 Department of Pediatrics, Kanti Children's Hospital, Maharjagunj, Kathmandu, Nepal; 2 Department of Pediatric Surgery, Kanti Children's Hospital, Maharjagunj, Kathmandu, Nepal; 3 Sangla Primary Health Center, Tarkeshwor, Kathmandu, Nepal; 4 Gajuri Primary Health Center, Dhading, Nepal; 5 Rapti Academy of Health Sciences, Ghorahi, Dang, Nepal

**Keywords:** *clinical profile*, *infectious diseases*, *pediatric hospital*, *respiratory disease*, *sociodemographic*

## Abstract

**Introduction:**

Child health is crucial in low and middle-income countries. Pediatric healthcare in tertiary-level hospitals addresses complex medical conditions. This study aimed to describe the sociodemographic and clinical profiles of pediatric patients admitted to Kanti Children's Hospital in Nepal.

**Methods:**

An observational cross-section study was conducted including all the inpatients under 15 years of age. The data were collected from the hospital medical record section from July 17, 2023 to July 15, 2024, after the ethical approval from Institutional Review Review Committee, (Reference number 2168). The statistical analysis included descriptive statistics to assess demographic characteristics, case types, admission patterns, and outcomes using Microsoft Excel and Statistical Package for Social Sciences (SPSS) 2024.

**Results:**

A total of 9682 pediatric cases were included, of which 6389 (65.99%) of the patients were male, with 3305 (34.13%) being aged 1 to 5 years. Among the admitted patietns 2194 (22.66%) had respiratory cause and 1520 (15.70%) had infectious disease. Mortallity rate was 203 (2.09%) and it was 82 (12.06%) in pediatric intensive care unit and 48(10.62%) in neonatal intensive care unit.

**Conclusions:**

Respiratory and infectious diseases were the most common cause of admission in pediatric settings with a higher prevalence in younger children. The majority of cases were residing outside the capital city. This study highlighted higher mortality rates in critical care units.

## INTRODUCTION

Child health is regarded as a crucial determinant of the nation's health care system.^1^ In low and middle-income countries, respiratory infections and diarrhea are leading causes of morbidity and mortality in children.^2^ Prompt medical intervention of such illnesses is critical in preventing child mortality.^3^

Tertiary-level pediatric hospitals provide specialized care and management.^4^ Admission patterns depend on referral sources, including general practitioners, other hospitals, and self-referrals.^5^ Global hospital admission rates continue to rise annually, resulting in increased demand for inpatient services.^6^ The change in hospital admissions among children can influence the workload of healthcare teams.^7^

There are limited data on the sociodemographic and clinical characteristics of pediatric inpatients in Nepal. This study primarily aimed to describe the sociodemographic and clinical profiles of admitted pediatric patients; their outcomes and admissions.

## METHODS

An observational cross-section study was conducted among the patients admitted to Kanti Children's Hospital over one year (July 17, 2023 - July 15, 2024) after approval was obtained from the Institutional Review Review Committee (Reference number 2168).

Data were obtained retrospectively from the medical record section. All in-patient cases from birth to the age of 15 years admitted to different wards; Medical ward, Surgical ward, Pediatric Intensive Care Unit (PICU), Neonatal Intensive Care Unit (NICU), Neonatal Intermediate Care Unit (NIMCU), Pediatric Intermediate Care Unit (PIMCU) Surgical Intensive Care Unit (SICU), Surgical High Dependency Unit, Oncology ward, Burn ward and Psychiatry ward of Kanti Children's Hospital during the study period, with complete records were included in the study. Cases with incomplete data in the hospital record system, and those treated and discharged from either emergency or observation wards were excluded from the study.

A total of 9719 cases were obtained from the medical record section, however, 37 cases that had incomplete data were excluded and thus 9682 cases were included in the study.

Descriptive analysis was carried out to describe the socio-demographic and clinical profile of the patients. The data were entered into a Microsoft Excel sheet. The statistical analysis was performed using IBM SPSS Statistics for Windows, Version 26( IBM Corp., Armonk, N.Y, USA).

## RESULTS

A total of 9682 cases were included in the study. The median age was 5.0 (IQR: 2.3-8.0 years). Among the admitted patients, 3305 (34.13%) were between 1 and 5 years, 2986 (30.84%) were between 5 and 15 years, 2199 (22.71%) were infants from 1 to 12 months, and 1192 (12.31%) were neonates. Out of 9682 patients, 6389 (65.99%) were males with male to female ratio of 1.94:1 (Table 1)

Among surgical cases, inguinal hernias accounted for 400 (20.79%), acute appendicitis for 269 (13.98%), hydrocele for 160 (8.31%), hypospadias for 153 (7.95%), Hirschsprung disease for 139 (7.22%), anorectal malformations for 121 (6.28%), and undescended testes for 83 (4.31%) admissions.

**Table 1:** Demographic characteristics of the study population (n=9682).

Indicators	n (%)
**Age group**	
1 to 15 years (Median (IQR))	5.0 (2.3-8.0)
<1 month	1192 (12.31)
1 to 12 months	2199 (22.71)
1 to 5 years	3305 (34.13)
5 to 15 years	2986 (30.84)
**Sex**	
Female	3293 (34.01)
Male	6389 (65.99)
**Ethnicity**	
Dalit	1357 (14.02)
Janajati	3458 (35.72)
Madhesi	909 (9.39)
Muslim	284 (2.93)
Brahmin/Chhetri	3674 (37.95)
**Residence**	
Within Kathmandu Valley	2979 (30.77)
Outside Kathmandu Valley	6703 (69.23)
**Types of cases**	
Medical	7200 (74.36)
Surgical	2482 (25.63)
**Admitted Ward**	
Medical	5258 (54.30)
Surgical	1924 (19.87)
Oncology	878 (9.07)
Neonatal (NICU+NIMCU)	1158 (11.96)
Neonatal Intermediate Care Unit (NIMCU)	706 (7.29)
Neonatal Intensive Care Unit NICU	452 (4.67)
Pediatric Intermediate Care Unit (PIMCU)	1583 (16.35)
Pediatric Intensive Care Unit (PICU)	680 (7.02)
Burn	151 (1.56)
Surgical Intensive Care Unit (SICU)	413 (4.27)
Surgical High Dependency Unit	252 (2.60)
Psychiatric	2 (0.02)

**Figure 1:**
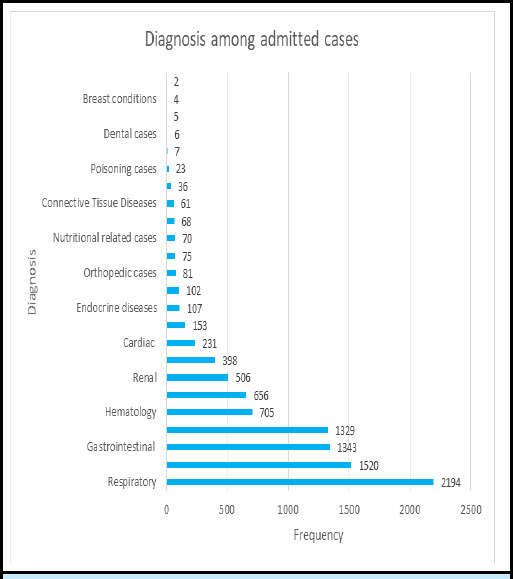
Diagnosis of Patients Admitted to Tertiary Level Pediatric Hospital (n=9682).

Among 9682 cases, 2194 (22.66%) cases were admitted because of respiratory cause, with diagnosis of pneumonia in 961 (43.80%) cases, acute bronchiolitis in 652 (29.71%) cases, and bronchial asthma in 69 (3.14%) cases. Infectious diseases accounted for 1520 (15.70%) cases, among 1211 (12.50%) were diagnosed as sepsis. (Figure 1).

A total of 203 (2.09%) mortality cases were recorded among 9682 admitted patients. The PICU had 82 (12.10%) deaths out of 680 admissions, and the NICU had 48 (10.40%) deaths out of 452 admissions. The Burn ward recorded 1 (0.70%) death out of 151 admissions (Table 2).

**Table 2:** Mortality among Patients Admitted to Tertiary Level Pediatric Hospital (N=9682).

Admitted Wards	Mortality	Total
PICU	82 (12.06)	680
NICU	48 (10.62)	452
SICU	39 (9.44)	413
Surgical HDU	2 (0.79)	252
Oncology	20 (2.28)	878
PIMCU	5 (0.32)	1583
Surgical	4(0.21)	1924
Medical	2 (0.08)	5258
Burn	1 (0.66)	151
NIMCU	-	706
Psychiatry	-	2
**Age Group**		
<1 month	77 (6.45)	1192
1 to 12 months	69 (3.13)	2199
1 to 5 years	29 (0.87)	3305
5 to 15 years	28 (0.93)	2986

PICU: Pediatric Intensive Care Unit; NICU: Neonatal Intensive Care Unit; SICU: Surgical Intensive Care Unit; Surgical HDU: Surgical High Dependency Unit; PIMCU: Pediatric Intermediate Care Unit; NIMCU: Neonatal Intermediate Care Unit.

**Table 3:** Outcome of Patients Admitted to Tertiary Level Pediatric Hospital (N=9682).

Discharge Status	n (%)
**Outcome**	
Recovered/Cured	9022 (93.18)
Leave against medical advice	242 (2.50)
Death	203 (2.09)
Referred	95 (0.98)
Discharge on request	67 (0.69)
Absconded	40 (0.41)
Not Improved	13 (0.13)
**Length of stay**	
<1 day	78 (0.81)
1 day	256 (2.64)
2 to 7 days	6676 (68.95)
>7 days	2672 (27.60)

A total of 242 (2.50%) patients left against medical advice, while 203 (2.09%) deaths were recorded. The length of stay had a median stay was 5.0 ( IQR: 3.0-8.0) among study patients.(Table 3)

**Figure 2:**
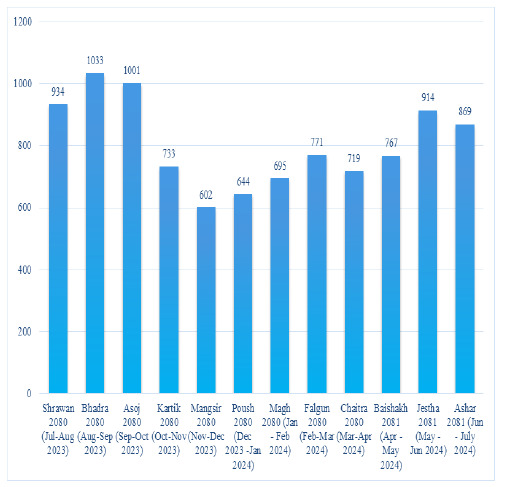
Monthly Admission trend at the hospital

Admissions in the first month of fiscal year 2080 Shrawan (July-August 2023) was 931 that peaked to 1033 in Bhadra 2080 (August-September 2023), the highest during the year. The lowest count of 602 in Mangsir 2080 (November-December 2023) was recorded. The data highlights fluctuations, with notable peaks in Bhadra 2080 and Jestha 2081 and the lowest in Mangsir 2080. (Figure 2)

The service parameters showed a doctor-to-patient ratio of 1:22.5, with 79 doctors available. The nurse-to-patient ratio was 1:55.0, supported by 175 nurses. The bed-to-patient ratio was 1:37.5, with a total of 258 beds available.

## DISCUSSION

Child health faces many challenges in developing countries like Nepal, where limited resources and infrastructure are important determinants for efficient pediatric healthcare delivery. According to the 2021 census, Nepal's total population is approximately 29.16 million, with about 27.8% (8.11 million) comprising children aged 0-14 years.^8^ It is essential to understand children's socio-demographic and clinical profiles requiring admission to a tertiary-level hospital to improve resource allocation within the healthcare system.

The mean age of patients in this study was 5.6 years, ranging from less than one month to 15 years. The findings also showed that the majority of patients seeking care at the pediatric inpatients were younger children, especially those between the ages of 1 and 5 years. A study conducted in a Teaching Hospital of Kathmandu, Nepal by Sharma AK et al. (2019) showed that the mean age of patients admitted during the study period was 5.6 years with a range of 1 month to 18 years.^9^ The result was quite similar to the result in this study. This correlates with the demographic distribution of Nepal where the majority of the people are below 15 years, further stressing the effectiveness of pediatric health care.

About 4.67% of the children were admitted to the neonatal intensive care unit. The prevalence of neonatal admission in the NICU was higher (40.6%) in a done at Pokhara, Nepal (2022).^10^ The contradiction might have occurred due to the nature of the sample as the present study included children under 15 years while the later study was focused on neonates.

The high admission rates as seen in the Medical Ward (54.30%) and the Pediatric Intermediate Care Unit (16.35%) indicate flexibility in the ability of the facility to address different cases. But the specialized units that include NICU and PICU has high mortality rate of 10.62% and 12.06% respectively which shows that critical care is difficult. These statistics raise issues in available NICU and PICU, which might be due to scarcity of resources, delayed admission, and patients with complicated conditions. The challenges could be reduced if there were increased access to new medical equipment and trainings for health service providers for better practice. The mortality rate was higher than the study conducted in Gandaki Medical College, Pokhara, Nepal (2021) with 3.46% deaths.^11^ The PICU mortality rate was lower than the study conducted in Kanti Children's Hospital, Kathmandu, Nepal, by Joshi P et al. (2019) with a mortality rate of 18.46%.^12^ This difference over five years period in a same hospital could be due to subsequent improvement in PICU in terms of availability of modern and increased number of medical equipment, trained and skilled health personals in critical care unit and change in clinical practice in due course of time. Furthermore, medical and surgical cases were 74.36 % and 25.63 % respectively. Medical cases were much higher than surgical cases which can be attributed to the increased burden of respiratory, infectious and gastrointestinal diseases as compared to surgical cases, this might reflect the impact of environmental factors like increasing pollution, lack of health education, inadequate health seeking behaviour and limited health facilities mainly in the peripheral and rural parts of Nepal. The prior history of inadequate treatment, delayed presentation, severity of the disease and increased length of stay are the factors that contribute to increased morbidity and mortality in ICUs as seen in other studies done in Egypt (2016) and Austria (2006).^13,14^

In the present study most of the admitted patient had, respiratory cases were the most common, accounting for 22.66%, followed by infectious disease (15.7%) and gastrointestinal cases (13.87%). These findings were consistent with another study at Bharatpur, Nepal (2015) which also showed that respiratory diseases (40.9%), followed by gastrointestinal diseases (22.2 %) were the most common reason for admission.^5^ Similarly, another study done at North India (2017) showed that the most common clinical indication for PICU admission were respiratory diseases (46.2%).^15^ The findings were consistent with another study from Nepal as well.^9^

In the current study, 2.50% patients left against medical advice, while (2.09%) deaths were recorded. Additionally, (0.98%) patients were referred to other facilities, and (0.69%) were discharged on request. Similar findings were observed in another study where the majority (89.6%) were discharged, 8.4% of patients left against medical advice, 2% were referred, and 0.1% were expired.^5^

This study highlighted the doctor-to-patient ratio which reflects the significant burden placed on healthcare providers in Nepal. This ratio indicates challenges in ensuring personalized and comprehensive care for each patient, particularly in a pediatric context where timely and detailed attention is important.^16^ Similarly, nurse to patient ratio indicates a considerable workload for nurses in critical care of NICU/PICU patients. The shortage of nurses likely contributes to challenges in providing adequate care which increases the risk of burnout among healthcare workers and affects patient outcomes as seen in a systematic review study by Tamata AT et al. (2023).^17^

The findings of this study underscore the urgency of addressing these challenges. With a high prevalence of respiratory and infectious diseases among pediatric admissions and concerning mortality rates in critical care units, the study reflects a scenario where resource constraints and the increasing demand for adequate pediatric healthcare. These outcomes also highlighted the urgent need for improved healthcare infrastructure and efficient resource allocation to ensure better health outcomes for children in Nepal.

This study also highlighted the monthly trends of admissions of 12 months duration. Admissions in the first month of fiscal year 2080 Shrawan (July-August 2023) was 931 that peaked to 1033 in Bhadra 2080 (August-September 2023), the highest during the year. The lowest count of 602 in Mangsir 2080 (November-December 2023) was recorded. The data highlights fluctuations, with notable peaks in Bhadra 2080 and Jestha 2081 and the lowest in Mangsir 2080. The reasons for fluctuations in admissions trends could be due to seasonal variations of certain diseases. This study also showed that more than two third (69.23%) of the in-patients were from outside the Kathmandu valley. This could be due two major reasons; people mentioning their permanent address outside Kathmandu despite residing temporarily in Kathmandu and other cause could be most of the patients were being referred from peripheral hospitals and health care centers and trust of people more in central hospitals.

This study has some limitations. In this study the age wise distribution of disease was not studied, which could have given us an idea about the prevalence of certain disease in certain age groups. Being a single-center study, the findings may not be generalizable to all pediatric hospitals of Nepal. The cross-sectional design limits our ability to establish causal relationships. Further research, including age wise distribution of diseases, multi-center studies and longitudinal designs, is needed to confirm these findings and explore the long-term impact of these factors on child health outcomes.

## CONCLUSIONS

Respiratory and infectious diseases were the most common cause of admission in pediatric settings with a higher prevalence in younger children. The majority of the cases were from outside the capital city.
